# Supplementary material for: Tryptophan-Derived Metabolites and Glutamate Dynamics in Fatal Insulin Poisoning: Mendelian Randomization of Human Cohorts and Experimental Validation in Rat Models
Source: Int J Mol Sci. 2025 Apr 27;26(9):4152. doi: 10.3390/ijms26094152 (PMC12072148; doi:10.3390/ijms26094152)
Supplement: Supplementary file 1 [file ijms-26-04152-s001.zip › Supplementary Figures.pdf]

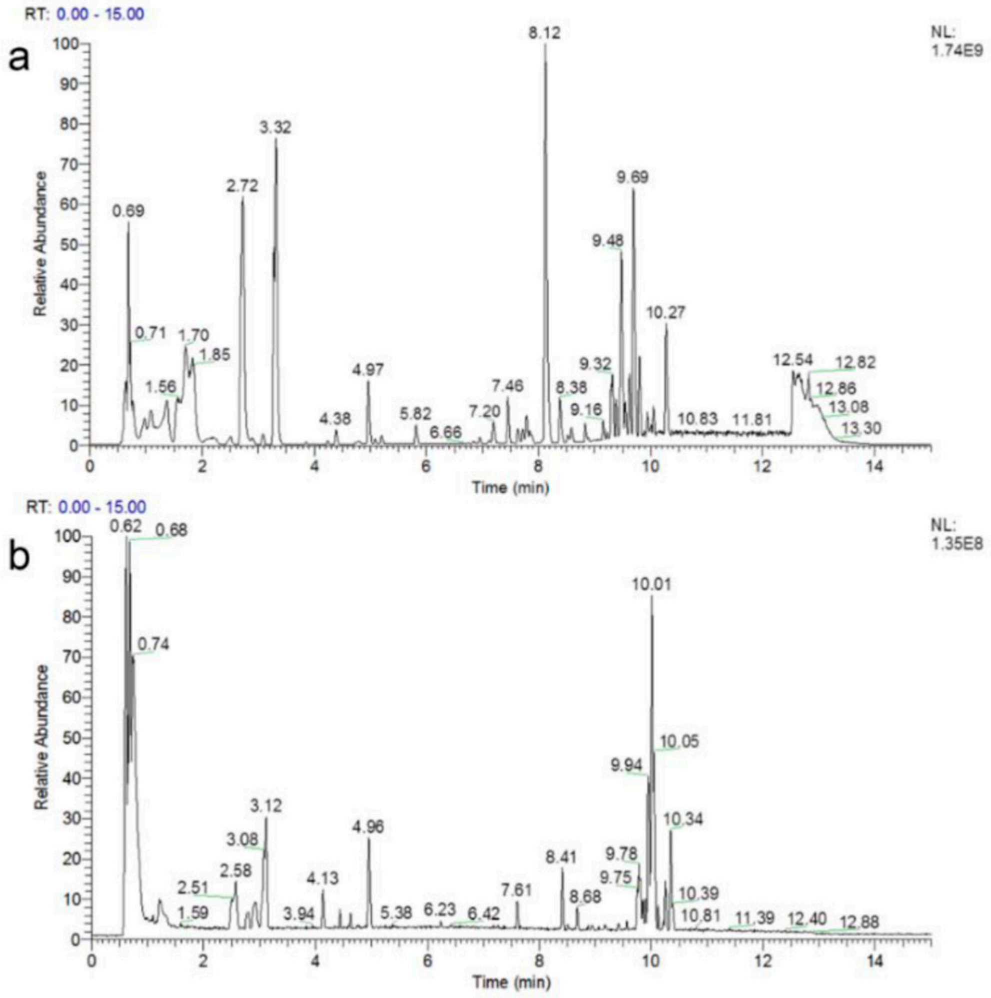

Supplementary Figure S1. (A) Overlaid BPCs of QC samples: (a) ESI+ mode; (b) ESI− mode.

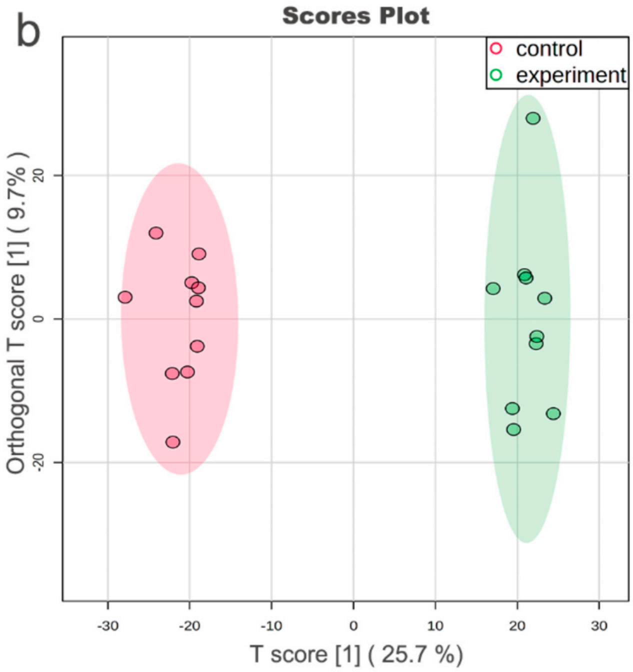

Supplementary Figure S2. OPLS-DA score plot of serum metabolomes
